# Molecular Epidemiology and Pathogenicity Evaluation of Porcine Teschovirus in Tibetan Pigs on the Qinghai–Tibet Plateau of China

**DOI:** 10.1155/tbed/8803438

**Published:** 2026-09-07

**Authors:** Haohao Lu, Min Zhang, Danjiao Yang, Xue Gao, Shuo Feng, Yiwen Pei, Zhidong Zhang, Long Zhou

**Affiliations:** ^1^ Key Laboratory of Veterinary Medicine in Universities of Sichuan Province, College of Animal and Veterinary Sciences, Southwest Minzu University, Chengdu 610041, China, swun.edu.cn; ^2^ Institute of Animal Science of Ganzi Tibetan Autonomous Prefecture of Sichuan Province, Kangding 626000, China; ^3^ Key Laboratory of Ministry of Education and Sichuan Province for Qinghai-Tibetan Plateau Animal Genetic Resource Reservation and Utilization, Chengdu 610041, China

**Keywords:** molecular epidemiology, pathogenicity, porcine diarrheal disease, porcine teschovirus, Tibetan pig

## Abstract

Porcine teschovirus (PTV) is the causative agent of porcine diarrhea and multisystem disorder and poses a global threat to the health of domestic pigs. However, its epidemic and pathogenic characteristics in Tibetan pigs, which are a unique indigenous breed in the Qinghai–Tibet Plateau of China, remain largely unexplored. Here, we conducted a comprehensive investigation during 2024–2025, in which 303 diarrheic fecal samples were collected from 21 farms across eight counties in Ganzi Tibetan Autonomous Prefecture, with an average altitude of 3433 m. RT‐PCR testing identified 141 PTV‐positive samples, yielding a high positivity rate of 46.5% (141/303). From these positives, 27 VP1 sequences were cloned and sequenced; phylogenetic analysis revealed that 16 strains belonged to *Teschovirus A*, three strains were divided into *Teschovirus B*, and three strains formed the interspecies recombinant genotypes (PTV‐15/16). Interestingly, five novel strains were classified as undefined genotypes, indicating an extensive genetic diversity among PTV strains circulating in Tibetan pigs. Furthermore, a PTV strain, designated PTV‐SCgz‐01, was successfully isolated in PK‐15 cells, with a near‐complete genomic sequence of 7081 nucleotides. Phylogenetic analysis based on the polyprotein and the VP1 genes indicated that it belonged to genotype PTV‐4, whereas recombination analysis revealed that PTV‐SCgz‐01 is a natural recombinant with parental strains derived from HNMY (PTV‐4) and China/SWU‐ZG2/2018 (PTV‐6). Experimental infection of 17‐day‐old Tibetan piglets demonstrated that this isolate induces severe watery diarrhea. Notably, the virus also caused severe pulmonary hemorrhage and mild cerebral hyperemia with neuronal degeneration, and it had a high mortality rate (40%), suggesting that strain PTV‐SCgz‐01 has strong pathogenic potential for 17‐day‐old Tibetan piglets. Our findings provide a more comprehensive molecular epidemiology of PTVs in Tibetan pigs and underscore the need for viral surveillance and control in this unique pig population.

## 1. Introduction

Porcine teschovirus (PTV) infection, also known as Teschen or Talfan disease, is a viral disease that can cause various disease syndromes, including diarrhea, polioencephalomyelitis, reproductive failure, pneumonia, and carditis [1–4]. The etiological agent of this disease, the PTV, is a nonenveloped virus that contains a single‐stranded, positive‐sense RNA genome of ~7.1 kb [5]. According to the International Committee on Taxonomy of Viruses (ICTV), PTV has been classified in the genus *Teschovirus* of the family *Picornaviridae*. The genome of PTV contains a single large open reading frame (ORF) flanked by 5′‐ and 3′‐untranslated regions (UTRs) [6]. The ORF codes for a leader protein (Lpro), four PTV structural proteins, including VP4, VP2, VP3, and VP1, and seven nonstructural proteins, including 2A, 2B, 2C, 3A, 3B, 3C, and 3D [7, 8].

PTV infection was first reported as a devastating pathogen in pigs in Teschen, Czechoslovakia, in 1929. In the subsequent 20 years, the disease spread rapidly to European countries, followed by the United States, Canada, Australia, and other continents. To date, the disease has spread widely all over the world, causing severe economic losses to the swine industry [9–12]. PTV genotyping is commonly based on VP1 sequence diversity as VP1 is the main antigenic structure and provides sufficient resolution for distinguishing recognized genotypes [9, 10, 13]. However, for newly emerged or recombinant PTV strains, VP1‐based genotyping alone may be insufficient for definitive classification. Therefore, additional genomic evidence, such as full‐length genome analysis and recombination detection, is required to refine the classification of novel strains. Currently, PTV is classified into two genotypes, genotype A contains 14 subgenotypes (PTV A1–A14), and genotype B contains three subgenotypes (*Teschovirus* B1–B3). Natural interspecies recombination events between PTV field strains have been described. Two PTV subgenotypes (PTV‐15 and PTV‐16) are interspecies recombinants between *Teschovirus A* and *Teschovirus B* [14–16]. In China, genotype A of PTV has circulated and predominated in the field since its initial emergence in 2003. According to the epidemiological investigation of PTVs during 2003–2026, genotype A of PTVs was detected in 12 provinces/cities, including Shandong, Hebei, Heilongjiang, Jilin, Tianjin, Henan, Inner Mongolia, Hunan, Shanghai, Guangxi, Jiangxi, and Gansu [4, 12–14, 17–23]. Genotype B of PTVs has only been identified in two provinces (Hunan and Jiangxi) [15, 16, 20]. However, PTV field strains have undergone continuous mutation and recombination, and several novel strains that are different from the known PTV genotypes have been discovered in recent years [14].

Domestic pigs and wild boars are the only known hosts of PTV [24]. In general, PTV infections are most often asymptomatic in pig herds; however, PTV variants with high pathogenicity have been reported in China in recent years. Tibetan pigs are a unique high‐altitude pig breed in China, mainly distributed in the Qinghai–Tibet Plateau, with an average altitude of about 3000 m [25]. In China, PTV infection cases and epidemiological studies have been primarily reported in domestic pigs. In 2018, a previous epidemiological survey detected PTV in Tibetan pigs from Kangding, with a positivity rate of 20.8% [26]. However, the prevalence and genetic diversity of PTV in Tibetan pigs across the broader Ganzi Prefecture remain largely unexplored. In this study, we conducted an extensive investigation of PTVs in fecal samples from Tibetan pigs on the Qinghai–Tibet Plateau, covering eight counties in Ganzi Prefecture. Furthermore, a PTV strain was isolated from diarrheic piglets on a pig farm in this region. Its genomic sequence was analyzed, and its pathogenicity was assessed.

## 2. Materials and Methods

### 2.1. Sample Information and PTV Detection

**Figure 1:**
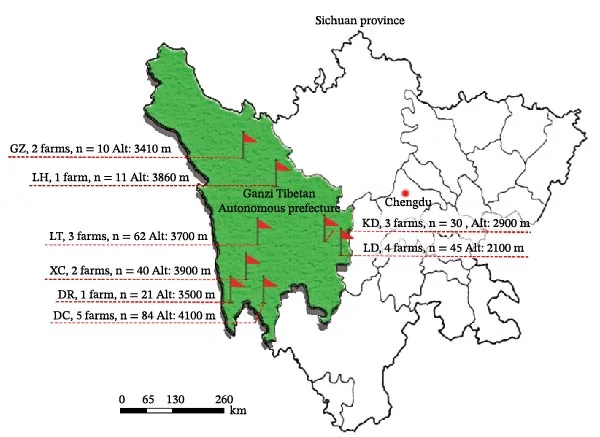
Geographical distribution of diarrheal sample collection sites in eight counties of Ganzi Tibetan Autonomous Prefecture, Sichuan Province. The altitude (Alt) of the sample collection sites ranges from 2100 to 4100 m.

From July 2024 to April 2025, a total of 303 fecal samples were collected from Tibetan piglets associated with the diarrhea disease. The samples originated from 21 farms across eight counties in the Ganzi Tibetan Autonomous Prefecture, Sichuan Province (Figure 1), including Luding (LD, four farms, *n* = 45), Litang (LT, three farms, *n* = 62), Daocheng (DC, five farms, *n* = 84), Xiangcheng (XC, two farms, *n* = 40), Kangding (KD, three farms, *n* = 30), Ganzi (GZ, two farms, *n* = 10), Derong (DR, one farm, *n* = 21), and Luhuo (LH, one farm, *n* = 11). The altitude of the fecal sample collection sites ranges from 2,100 to 4,100 m, with an average altitude of 3,433 m. These samples were collected using separate containers and individual polyester‐tipped swabs for each animal. PTV initial screening among the 303 clinical samples was performed using a primer pair (forward: 5′‐TGTTGTGTTTAAACACAGAAAT‐3′ and reverse: 5′‐TTCAACTGACTATACAAAGTAC‐3′) that amplifies a 307 bp fragment. Furthermore, the complete sequences of the VP1 gene from PTV‐positive samples were amplified and sequenced using the nested PCR primer pairs previously reported by Yang et al. [20].

### 2.2. Cells and Virus Isolation

Virus isolation was performed in porcine kidney cells (PK‐15). PTV‐positive samples were filtered through 0.22 µm membranes (Millipore, United States). Then, 500 µL of the filtrate was inoculated onto monolayer PK‐15 cells in 6‐well plates and adsorbed for 1.5 h at 37°C. Then, the inoculum was discarded, and DMEM supplemented with 1% fetal bovine serum (FBS) was added. Blind passages in PK‐15 cells were carried out until the cytopathic effect (CPE) was observed. The culture was subjected to three freeze–thaw cycles, followed by centrifugation at 8000 ×*g* for 10 min at 4°C to pellet the cell debris, and the resulting supernatant was stored at −80°C. In addition, the virus was further purified twice by plaque assay as previously described [12], and then, the purified virus was used for viral identification and whole‐genome sequencing.

### 2.3. Immunofluorescence Assay (IFA)

For the IFA, PK‐15 cells seeded in 6‐well plates and infected with PTV were fixed with 4% paraformaldehyde for 20 min at room temperature. The fixed cells were then permeabilized with 0.2% Triton X‐100 and blocked with 5% BSA for 1 h. Subsequently, the cells were incubated with a rabbit anti‐PTV‐VP1 polyclonal antibody (1:200 dilution) for 2 h, followed by a FITC‐conjugated goat anti‐rabbit IgG secondary antibody (1:200 dilution; Biosharp, China) for 1 h at 37°C. Cell nuclei were counterstained with DAPI (Beyotime, China). Images were acquired with a fluorescence microscope.

### 2.4. Viral Replication Kinetics in PK‐15 Cells

PK‐15 cell monolayers in 6‐well plates were infected with the PTV‐SCgz‐01 strain at an MOI of 0.1. Following a 2‐h adsorption period, the inoculum was removed, and the cells were washed three times with PBS before the addition of 2 mL of maintenance medium. To quantify virus production, supernatants were harvested at 12‐h intervals for 72 h postinfection, and viral titers were determined by the TCID_50_ assay. Viral titers were calculated using the Reed–Muench method, and the viral growth curve was generated with GraphPad Prism 10 software.

### 2.5. Electron Microscopy

PTV‐infected cells were harvested for virion imaging. The culture supernatant was centrifuged at 12,000 ×*g* for 2 h to remove cellular debris. The resulting virion pellet was resuspended in PBS, negatively stained with 2% phosphotungstic acid, and visualized using a JEM‐1400FLASH transmission electron microscope (TEM) (JEOL, Japan).

### 2.6. Virus Genome Amplification and Sequencing

Viral RNA was extracted from samples of clarified infected cell lysate using TRIzol and reverse transcribed using the TaKaRa One Step RT‐PCR Kit with random primers. The near‐complete genome was amplified in nine overlapping fragments using specific primer pairs (Table 1). The resulting PCR products were cloned into the pMD19‐T vector (TaKaRa, Dalian, China) and subjected to Sanger sequencing by Sangon Biotech (Shanghai, China). The genome sequence was assembled using DNAstar software (Version 7.0), and the assembled sequence of the PTV‐SCgz‐01 strain has been deposited in the GenBank database under Accession Number PX625739. The exact 5′ and 3′ termini were not experimentally determined by the RACE.

**Table 1:** The primers used for near‐complete genome amplification of PTV isolate in this study.

Primer	Nucleotide sequences (5′→3′)	Position
PTV‐1F	GGTAAGTGATGATCCTTGGAAA	1–22
PTV‐1R	TTGGTGCAATACCTGGTGAC	180–199
PTV‐2F	TATCTTGTGCCCAACATTAGTCC	52–74
PTV‐2R	CTCCAGTATTCCCAGAGTTATTTGT	689–713
PTV‐3F	TCTCCTGCGTCTTGAAACAT	517–536
PTV‐3R	AACACCCAAACATCCTCCA	1231–1249
PTV‐4F	TGCCCTATGCTCTTGCTACT	1119–1138
PTV‐4R	TTTTGTGCTGGACACTTCTTG	2603–2623
PTV‐5F	TGCGTCAAATGAAAACCCT	2506–2524
PTV‐5R	TGTATTGGACCCTGGAAGTG	3725–3744
PTV‐6F	GTGGATTCTGGAGCCGTTTT	3696–3715
PTV‐6R	CCATTGTGCTTCTCATAAGTCATT	5107–5130
PTV‐7F	CAAAACCCAACAGGCAATC	5001–5019
PTV‐7R	GCCGACCGAATAACATCC	6222–6239
PTV‐8F	AGCAGCCCTTTCACATCAT	5758–5776
PTV‐8R	GAACATTTCACGCCACTCC	6994–7012
PTV‐9F	CTCCCACGCCAGGCTTAC	6911–6928
PTV‐9R	GCTCAACTAAAACTACTTGGGCA	7059–7081

### 2.7. Sequence and Phylogenetic Analysis

All available PTV reference sequences were retrieved from the GenBank database. A multiple sequence alignment was performed, and phylogenetic relationships were inferred using MEGA X software. Specifically, a phylogenetic tree based on the VP1 gene was constructed by the maximum likelihood (ML) method with 1,000 bootstrap replicates. The MegAlign tool (DNASTAR, Madison, WI, USA) was used to analyze the nucleotide (nt) and inferred amino acid (aa) sequences. Fifty PTV strains were acquired from the NCBI database to compare the genomic features of the PTV‐SCgz‐01 strain with those of previous PTV strains.

### 2.8. Recombinant Analysis of PTV‐SCgz‐01 Strain

Potential recombination events within the PTV‐SCgz‐01 genome were evaluated using Recombination Detection Program 4 (RDP4, v4.9.6) and SimPlot software (v3.5.1, Baltimore, MD, USA). RDP4 analysis was performed using seven detection methods (RDP, GENECONV, BootScan, MaxChi, Chimaera, SiScan, and 3Seq) with default parameters, and the potential recombination breakpoints predicted by RDP4 were further verified using SimPlot (v3.5.1).

### 2.9. Pathogenicity Analysis of the Isolated PTV‐SCgz‐01 Strain

Ten 14‐day‐old Tibetan piglets were obtained from a farm with no history of diarrhea. Prior to the study, all animals were confirmed by RT‐PCR to be negative for PTV, porcine rotavirus (PoRV), porcine epidemic diarrhea virus (PEDV), transmissible gastroenteritis virus (TGEV), and porcine deltacoronavirus (PDCoV). Meanwhile, the viral inoculum was tested to be free of PoRV, PEDV, TGEV, and PDCoV by RT‐PCR and bacterial contamination by sterility test. The piglets were randomly allocated into two groups (*n* = 5 per group) and housed in separate isolation rooms with a milk substitute diet. Following a 3‐day acclimatization period, during which the piglets reached 17 days of age, group A was orally inoculated with 5 mL of virus suspension containing 1 × 10^8.61^ TCID_50_/mL of the PTV‐SCgz‐01 isolate, corresponding to a total challenge dose of 5 × 10^8.61^ TCID_50_ per pig, while the control group B received an equal volume of uninfected DMEM. Postchallenge, piglets were monitored daily for 7 days for clinical signs. Diarrhea severity was scored using a 0–3 scale: no diarrhea = 0, sticky feces = 1, loose feces = 2, and watery feces = 3. Rectal swabs were collected every 24 h to measure viral load by reverse‐transcription quantitative PCR (RT‐qPCR). For the piglets that died prematurely during the experiment, rectal fecal swabs were collected and stored at −20°C until the analysis. All surviving piglets were humanely euthanized at 7 days postinoculation (dpi). Necropsy was performed immediately, and tissue samples (including brain, heart, liver, spleen, lung, kidney, small intestine, and large intestine) were collected and fixed in 4% paraformaldehyde for 48 h for subsequent histopathological examination by hematoxylin and eosin (H&E) staining and immunohistochemistry (IHC) using an anti‐PTV‐VP1 polyclonal antibody. The animal studies were approved by the Animal Care and Use Committee of Southwest Minzu University, Chengdu, Sichuan. This study was conducted in accordance with local legislation and institutional requirements, and written informed consent was obtained from all animal owners.

### 2.10. Real‐Time PCR Analysis Quantification

To quantify the PTV load in various tissues and fecal samples, total RNA was extracted and reverse transcribed into cDNA using the TaKaRa One‐Step RT‐PCR Kit with random primers. RT‐qPCR was subsequently performed using Archimed Analyzer Software (RocGene, China). The reactions were incubated at 95°C for 3 min, followed by 40 cycles of 95°C for 5 s and 60°C for 30 s. Each sample was run in triplicate. Based on the standard curve, the number of total viral RNA copies per gram of tissue was calculated.

## 3. Results

### 3.1. RT‐PCR Survey of PTV in Tibetan Pigs

Of the 303 fecal samples from the Tibetan pigs (age ranging from 1 day to 3 months) with diarrhea, 141 fecal samples were detected as positive for PTV using conventional RT‐PCR, resulting in a positivity rate of 46.53% (141/303). Among them, Ganzi showed the highest PTV positivity rate (70.0%; 7/10), followed by Litang (62.9%; 39/62), Luding (51.1%; 23/45), Xiangcheng (50.0%; 20/40), Daocheng (48.8%; 41/84), and Kangding (36.7%; 11/30). However, no PTV was detected in Derong (0%; 0/21) or Luhuo (0%; 0/11) (Table 2). In addition, PTV was detected positive in 19 pig farms across six counties with a positive rate of 90.5% (19/21), indicating a high prevalence and wide distribution of PTV in the surveyed region.

**Table 2:** Information and PTV detection of the fecal samples from the Tibetan pigs.

Collection site	Number of samples	Number of pig farms	Age of pigs	Number of PTV positives	Positive rate (%)
Luding	45	4	5–32 days	23	51.1
Litang	62	3	15 days−3 months	39	62.9
Daocheng	84	5	1 day−2 months	41	48.8
Xiangcheng	40	2	14 days−2 months	20	50.0
Kangding	30	3	14–45 days	11	36.7
Ganzi	10	2	≈35 days	7	70.0
Derong	21	1	10–37 days	0	0.0
Luhuo	11	1	5 days−2 months	0	0.0
Total	303	21	1 day−3 months	141	46.5

### 3.2. Phylogenetic Analysis of the VP1 Gene

**Figure 2:**
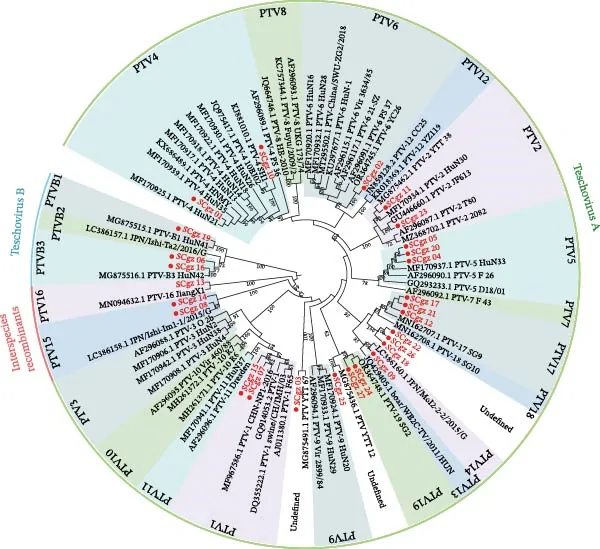
Phylogenetic analysis of the PTV isolates. A maximum likelihood (ML) phylogenetic tree was constructed based on the VP1 gene sequences. The analysis, performed using MEGA X with 1000 bootstrap replicates, reveals the genetic relationships among the isolates, with those identified in this study being highlighted in red.

Of the 141 PTV‐positive samples, 27 complete VP1 gene sequences were successfully cloned and sequenced. These strains were designated as SCgz‐01 to SCgz‐27 (GenBank Accession Numbers PX625740–PX625766). Based on the phylogenetic analysis of the complete VP1 sequences, 16 PTV strains were divided into eight subgenotypes of genotype A. Among these strains, SCgz‐07 and SCgz‐15 belonged to PTV‐1; SCgz‐11 and SCgz‐23 belonged to PTV‐2; SCgz‐01 and SCgz‐10 belonged to PTV‐4; SCgz‐04, SCgz‐05, and SCgz‐20 belonged to PTV‐5; SCgz‐02 belonged to PTV‐6; SCgz‐09 belonged to PTV‐14; SCgz‐12, SCgz‐17, and SCgz‐21 belonged to PTV‐17; and SCgz‐24 and SCgz‐27 were closely related to PTV‐19. Three PTVs were classified as genotype B, with SCgz‐19 belonging to B1 and SCgz‐06 and SCgz‐16 belonged to B3. Three PTVs were clustered into the interspecies recombinants: SCgz‐08 and SCgz‐14 belonging to PTV‐15 and SCgz‐13 belonging to PTV‐16. Notably, 5 PTVs (SCgz‐03, SCgz‐18, SCgz‐22, SCgz‐25, and SCgz‐26) were grouped as undefined genotypes (Figure 2). Multiple sequence alignment analyses showed that the nucleotide and amino acid homologies between the five undefined genotypes and other recognized PTV strains were nt 52.0%–73.1% and aa 27.7%–52.0%, respectively (Figure S1). Additionally, several unique amino acid mutation sites were identified in the five undefined genotype strains—such as 19E, 53A, 108G, 174T, 201Y, 216I, and 229I in the SCgz‐03 strain; 51L, 73L, 74E, and 204A in the SCgz‐25 strain; and 18Q, 173T, and 233Y in the SCgz‐18, SCgz‐22, and SCgz‐26 strains (Figure S1). The results indicated a high diversity of PTV genotypes in Tibetan pigs.

### 3.3. Virus Isolation, Identification, and Growth Curves of PTV Strains

**Figure 3:**
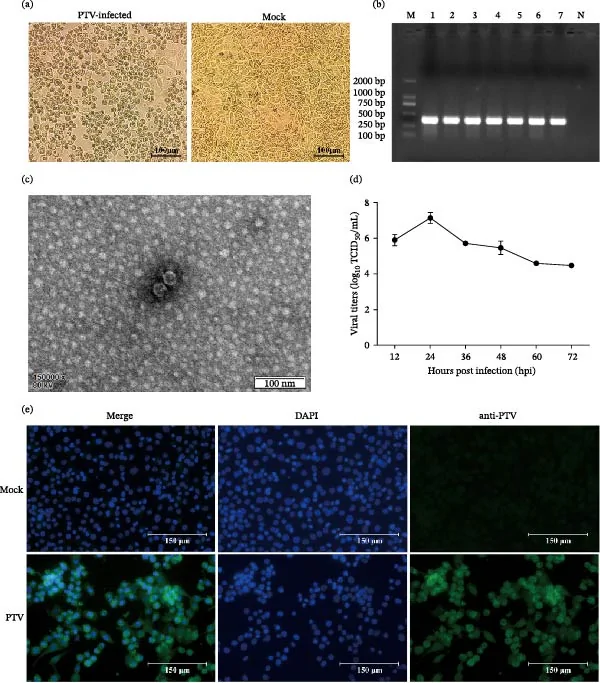
Isolation and characterization of PTV‐SCgz‐01. (a) Cytopathic effect in PTV‐SCgz‐01‐infected and mock‐infected PK‐15 cells. (b) RT‐PCR detection of different passages to the PTV. M: DL2, 000 DNA marker; Lanes 1–7: P1–P7, N: Negative control. (c) Transmission electron microscopic image of purified PTV particles. (d) Growth kinetics of PTV‐SCgz‐01 in PK‐15 cells. (e) IFA analysis of PTV‐infected cells and mock‐treated cells.

For virus isolation, the fecal suspensions of the 27 PTV‐positive samples were inoculated onto PK‐15 cells. After five blind passages in PK‐15 cells, only one sample showed obvious CPE and tested positive for PTV by RT‐PCR. The infected PK‐15 cells exhibited cell rounding and cluster‐like forms and eventually detached completely (Figure 3a). RT‐PCR further revealed that PTV remained detectable after serial passages, suggesting successful virus isolation (Figure 3b). The isolate, named PTV‐SCgz‐01, was further purified and passaged in PK‐15 cells for 10 passages and subjected to electron microscope observation. Spherical viral particles of ~30 nm in diameter were observed, and the features are consistent with the typical morphology of *Picornavirales* (Figure 3c). The growth characteristics of PTV were also described in PK‐15 cells with an MOI of 0.1. The result revealed that the isolate was adapted to cells, with the viral titers peaking at 24 h postinfection (hpi) (Figure 3d). Specific green fluorescence was observed in PK‐15 cells infected with the PTV‐SCgz‐01 strain by using an IFA (Figure 3e). No green fluorescence was observed in the mock cells. The results indicated that PTV‐SCgz‐01 was successfully isolated using PK‐15 cells, and the viral titer of PTV‐SCgz‐01 was determined to be 1 × 10^8.61^ TCID_50_/mL.

### 3.4. Genomic Sequence Analysis of PTV‐SCgz‐01

Because the exact 5′ and 3′ termini were not experimentally determined by RACE, the assembled sequence of PTV‐SCgz‐01 should be considered a near‐complete genome. The assembled genome sequence of the isolate was 7,081 nucleotides in length, with a 44.1% GC content. To characterize its genetic variation, the nucleotide sequences and deduced amino acid sequences of its P1, P2, P3, and polyprotein genes were aligned against 50 reference strains, which were classified into three species: *Teschovirus A*, *Teschovirus B*, and interspecies recombinants. The results demonstrated that the polyprotein gene of PTV‐SCgz‐01 shared the highest nucleotide identity (93.2%) with the HuN21 strain, which was isolated in Hunan Province in 2016. In contrast, the nucleotide identities between PTV‐SCgz‐01 and reference genomes of interspecies recombinants and *Teschovirus B* were relatively low, at 73.4%–74.6% and 71.2%–71.4%, respectively. With respect to individual gene regions, the nucleotide identity between PTV‐SCgz‐01 and *Teschovirus A* was 70.5–95.7% (corresponding to 68.0%–95.5% amino acid identity) in the P1 region, 84.0%–91.7% (94.1%–99.4% amino acid identity) in the P2 region, and 85.4%–97.3% (96.4%–99.0% amino acid identity) in the P3 region (Table 3). Notably, these identity values were consistently higher than those observed between PTV‐SCgz‐01 and the reference strains of interspecies recombinants or *Teschovirus B* strains.

**Table 3:** Nucleotide (nt) and amino acid (aa) sequence identity values for different regions compared with other PTV reference strains.

Species	Genotype	GenBank number	Strain	Structural proteins	Nonstructural proteins		Polyprotein
				P1 (nt/aa)	P2 (nt/aa)	P3 (nt/aa)	L + P1 + P2 + P3 (nt/aa)
*Teschovirus A*	1	AJ011380.1	F65	71.6/68.8	84.4/95.9	86.7/96.4	80.7/80.6
	1	DQ355222.1	Swine/CH/IMH/03	71.4/68.6	85.5/94.7	86.7/96.8	80.8/80.8
	1	MF967586.1	CHN‐NP1‐2016	71.9/68.3	89.8/97.1	91.5/98.4	83.7/83.7
	2	GU446660.1	JF613	78.4/77.7	88.6/98.2	89.8/98.7	85.0/92.3
	2	MZ368702.1	2082	79.5/77.6	85.7/94.9	88.6/98.1	84.5/92.4
	2	AF296087.1	T80	79.6/77.5	84.4/95.9	85.6/97.2	83.4/92.2
	3	MF170942.1	HuN38	72.4/69.6	91.2/98.6	89.0/97.5	83.1/83.0
	3	MF170906.1	HuN2	72.5/69.5	91.1/98.6	88.9/97.7	83.1/83.0
	3	AF296088.1	O 2b	72.7/69.3	84.8/95.3	85.4/96.9	80.7/80.7
	4	AF296089.1	PS 36	82.5/80.9	85.4/95.9	86.0/97.2	84.6/93.2
	4	KJ881010.1	SH1	81.7/81.8	88.5/97.7	91.9/98.4	86.9/94.1
	4	MF170925.1	HuN21	**95.7**/**95.5**	**91.7**/**99.4**	90.8/**99.0**	**93.2**/**99.1**
	4	MF170922.1	HuN18	92.8/92.7	87.8/97.3	90.4/98.7	90.9/98.0
	4	MF170917.1	HuN13	93.6/93.5	88.3/94.3	89.4/98.7	90.9/97.3
	4	MF170939.1	HuN35	86.6/86.5	90.0/98.6	**97.3**/**99.0**	91.4/95.2
	4	KX686489.1	HNMY	93.7/93.9	87.6/97.1	91.3/98.4	91.3/97.5
	4	JQ975417.1	10BJ02	82.3/81.8	90.6/98.8	90.2/98.3	87.6/95.0
	5	AF296090.1	F 26	73.5/70.7	85.3/95.3	86.2/97.0	81.3/67.3
	5	MF170937.1	HuN33	74.3/70.8	89.3/**99.4**	90.6/98.6	83.7/73.2
	5	GQ293233.1	D18/01	72.8/70.8	84.8/96.5	87.0/97.9	81.2/67.7
	6	AF296091.1	PS 37	80.2/77.6	84.9/95.5	86.6/97.0	84.0/92.2
	6	AF296115.1	Vir 3634/85	80.0/76.7	85.3/95.9	88.8/98.4	84.7/92.4
	6	MF170920.1	HuN16	79.4/78.7	89.1/97.7	90.3/98.7	85.8/93.2
	6	MT295502.1	PTV‐China/SWU‐ZG2/2018	80.3/79.2	88.6/98.6	89.5/98.6	86.1/94.0
	6	KU297677.1	HuN‐1	79.5/77.9	85.1/96.1	91.6/**99.0**	85.2/92.8
	6	AF296117.1	21‐SZ	79.8/77.1	84.0/94.7	90.5/98.3	85.0/92.1
	7	AF296092.1	F43	73.1/69.9	85.2/96.1	86.6/97.2	81.3/89.5
	8	JQ664746.1	HB‐2010	78.1/75.8	86.5/95.9	89.3/97.8	84.4/91.3
	8	KC757344.1	Fuyu/2009/2	79.8/79.2	86.3/96.1	89.9/98.7	85.2/93.2
	8	AF296093.1	UKG 173/74	79.4/78.5	84.5/95.7	86.3/96.6	83.4/92.1
	9	MF170924.1	HuN20	71.9/68.5	86.3/95.7	89.7/98.4	82.2/89.3
	9	MF170933.1	HuN29	71.7/68.9	88.4/98.0	90.1/98.6	82.5/89.7
	9	AF296094.1	Vir 2899/84	72.0/68.5	85.1/96.3	87.9/97.8	81.1/88.9
	10	AF296095.1	Vir 460/88	70.6/68.0	85.8/96.9	88.1/98.1	81.0/89.0
	10	MH261371.1	R6	70.5/68.2	85.3/95.7	87.5/97.7	80.4/88.2
	10	MH261372.1	R7	70.5/68.2	85.2/95.7	87.6/97.7	80.4/88.2
	11	MF170941.1	HuN37	71.3/68.9	86.3/97.1	88.8/98.1	81.4/89.3
	11	AF296096.1	Dresden	71.3/69.2	84.7/94.1	87.9/96.5	80.8/88.2
	12	KR018369.1	YZ119	79.7/77.9	86.1/96.5	89.3/98.2	85.0/92.8
	12	JN859128.2	CC25	79.8/78.1	85.8/96.3	86.7/97.4	84.0/92.3
	13	JQ429405.1	Boar/WB2C‐TV/2011/HUN	72.8/69.6	85.7/95.7	87.5/96.9	81.5/81.5
	14	LC386160.1	JPN/MoI2‐2‐2/2015/G	73.0/69.3	—	—	—
	17	MN162707.1	SG9	73.3/70.2	89.8/98.2	90.8/98.6	83.9/83.8
	18	MN162708.1	SG10	72.1/69.9	84.7/95.3	91.5/98.4	82.5/82.4
	19	OR364748.1	SG2	71.3/68.8	90.4/97.7	90.1/98.4	82.8/82.9
Interspecies recombinants	15	LC386158.1	JPN/Ishi‐Im1‐1/2015/G	70.8/67.9	80.1/90.0	74.4/83.3	74.6/74.8
	16	MN094632.1	JiangX1	67.7/63.9	79.8/89.5	74.7/83.1	73.4/73.4
*Teschovirus B*	B1	MG875515.1	HuN41	62.2/56.1	80.9/90.0	74.5/82.9	71.4/76.4
	B2	LC386157.1	JPN/Ishi‐Ta2/2016/G	62.0/56.5	80.1/89.8	74.7/83.1	71.2/76.6
	B3	MG875516.1	HuN42	63.0/57.5	79.8/90.0	74.4/82.8	71.4/76.8

*Note:* The highest nucleotide and amino acid identities are indicated in bold typeface.

### 3.5. Recombination Analyses of the PTV‐SCgz‐01 Isolate

**Figure 4:**
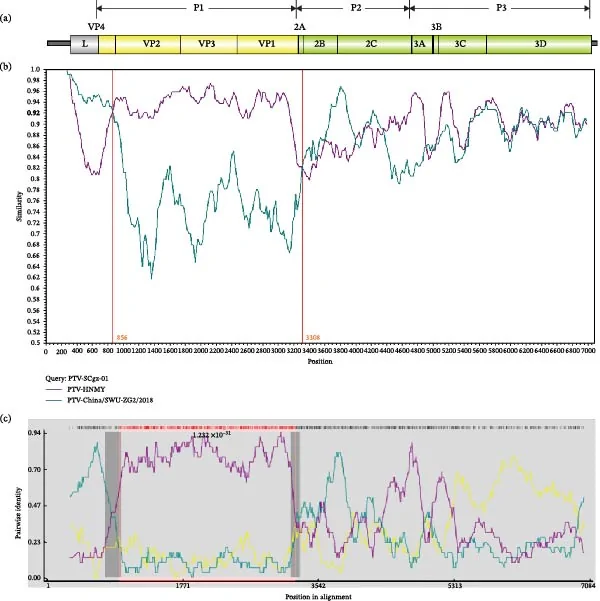
Genome recombination analyses of PTV‐SCgz‐01. (a) The genome schematic diagram of PTV: the yellow area indicates the region of structural protein, and the green area indicates the region of nonstructural protein. (b) Similarity plot analysis of the genome sequences of PTV‐HNMY (purple curve), PTV‐China/SWU‐ZG2/2018 (green curve), and PTV‐SCgz‐01 as query sequences; the *y*‐axis presents the percent identity with a window size of 200 bp and a step size of 20 bp. The vertical red line indicates the potential recombination breakpoint. (c) The recombination breakpoint analysis of PTV‐SCgz‐01 vs. PTV‐HNMY (purple curve), PTV‐SCgz‐01 vs. PTV‐China/SWU‐ZG2/2018 (green curve).

We examined the sequence alignment between SCgz‐01 and reference PTV strains using RDP4 and SimPlot to identify potential recombination events. Recombination analysis of the near‐complete genome revealed a possible recombination event primarily located in the P1 region (Figure 4a). SCgz‐01 exhibited high nucleotide sequence similarity with the PTV strain HNMY (KX686489) in the VP2, VP3, VP1, and 2A regions, while the remaining gene segments (L, VP4, and 2B to 3D) showed high similarity to PTV‐China/SWU‐ZG2/2018 (MT295502) (Figure 4b,c). RDP4 analysis indicated that SCgz‐01 is a natural recombinant virus originating from two parental strains, supported by highly significant *p*‐values (≤2.914 × 10^−08^), with detection by at least six methods: RDP, GENECONV, BootScan, MaxChi, Chimaera, and 3Seq (Table 4). Therefore, SCgz‐01 is likely a naturally recombinant virus derived from the recombination between PTV‐4 (HNMY) and PTV‐6 (China/SWU‐ZG2/2018).

**Table 4:** Information on recombination events detected in SCgz‐01.

Methods	Number of seqs detected in	Av. *p*‐value
RDP	1	1.232 × 10^−31^
GENECONV	1	1.682 × 10^−19^
BootScan	1	3.736 × 10^−27^
MaxChi	1	8.214 × 10^−24^
Chimaera	1	2.822 × 10^−18^
SiScan	—	—
3Seq	1	2.914 × 10^−08^

### 3.6. Pathogenicity of the PTV‐SCgz‐01 on Suckling Piglets

**Figure 5:**
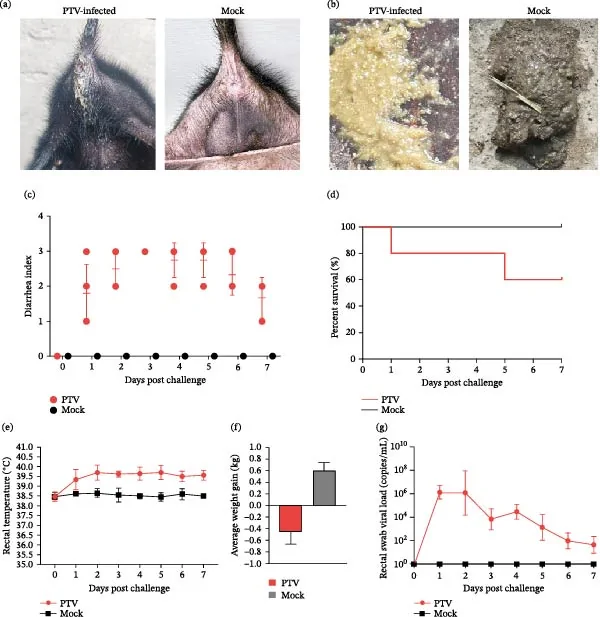
Clinical manifestations of PTV‐SCgz‐01 infection in Tibetan piglets. 17‐day‐old piglets were orally inoculated with 5 mL of PTV‐SCgz‐01 (*n* = 5) or 5 mL of DMEM as a mock control (*n* = 5). (a, b) Clinical manifestation of acute watery diarrhea in PTV‐challenged piglets at 2 dpi. (c) Clinical diarrhea index was evaluated in the PTV‐challenged and control groups from 0 to 7 dpi. (d) Survival curves of the inoculated pigs. (e) Rectal temperature (°C) of piglets in the PTV‐challenged and control groups from 0 to 7 dpi. (f) Average weight gain from days 0 to 7 postchallenged. (g) Dynamics of PTV RNA shedding in rectal swabs from the challenged piglets at all sampled time points during the 1–7 dpi observation period. After the death of two piglets at 2 and 5 dpi, no further samples were available from those animals.

During the challenge period, one piglet died at 2 dpi, and another piglet died at 5 dpi with severe watery diarrhea. The remaining three piglets in the challenge group exhibited emaciation and severe dehydration and were euthanized at 7 dpi (Figure 5d). Compared with the control group, the challenged piglets showed marked diarrhea and paralysis of the hind limbs (Figure 5a,b). The diarrheal scores showed a progressive increase. Subsequently, a gradual decrease was observed after 6 dpi (Figure 5c). The piglets developed a mild fever on 2 dpi, with body temperatures between 39.5 and 40.0°C (Figure 5e). The body weight of challenged piglets decreased since viral infection, while the control kept stable growing (Figure 5f). Viral RNA was detected in rectal swabs throughout the 1–7 dpi observation period, with sample availability decreasing after the death of two piglets at 2 and 5 dpi. Shedding peaked between 1 and 4 dpi, with titers ranging from 2.28 × 10^6^ to 5.11 × 10^4^ genome copies/mL, after which a decline was observed (Figure 5g).

**Figure 6:**
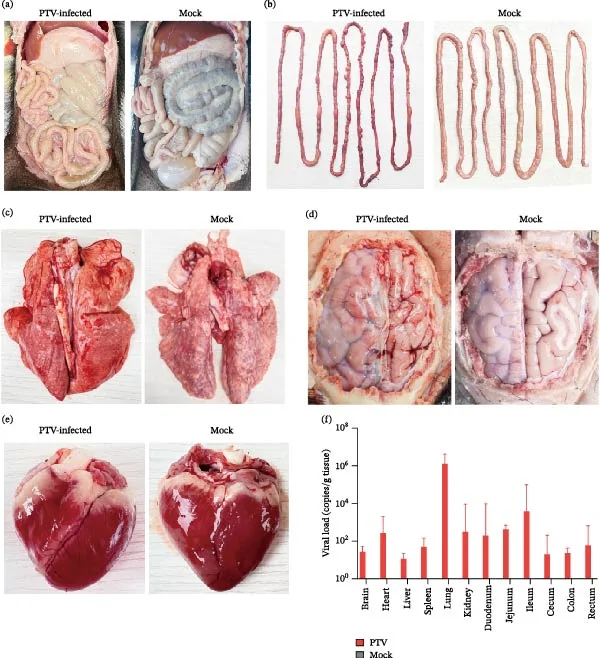
Gross pathology and viral distribution of PTV‐SCgz‐01 infection in Tibetan piglets. (a) Comparison of the intestines from PTV‐challenged and mock‐treated piglets at postmortem examination. (b) Representative gross images of the duodenum, jejunum, and ileum from a PTV‐challenged piglet, showing extensive hemorrhages throughout all three segments. (c, d) Gross pathology was notable for extensive hemorrhagic lesions in the lungs and brain following PTV challenge. (e) Heart of infected piglet, dark red with dilated epicardial vessels and congestion. (f) Viral load in various organs of PTV‐infected piglets at 7 dpi.

Postmortem examination revealed macroscopic lesions in the challenged group, such as thin and translucent intestinal walls and intestines filled with a yellowish watery fluid (Figure 6a). We observed mild hemorrhaging in the small intestine and mesentery of the infected piglets (Figure 6b). The lungs exhibited severe hemorrhage (Figure 6c). Brain necropsy examinations of the piglets were performed, and mild hyperemia was observed in the cerebrum of infected piglets (Figure 6d). The heart of infected piglets appeared dark red, with dilated epicardial vessels and generalized congestion (Figure 6e). At 7 dpi, piglets were euthanized, and viral load was quantified in various tissues. The PTV‐SCgz‐01 strain exhibited a broad tissue tropism, as evidenced by RT‐qPCR detection in most collected tissues, including the brain, heart, liver, spleen, lungs, kidneys, and all segments of the small and large intestines (duodenum, jejunum, ileum, cecum, colon, and rectum). The highest viral loads were found in the lungs, small intestine, kidneys, and heart (4.88 × 10^2^ to 2.18 × 10^6^ copies/g), while the liver contained relatively lower titers (Figure 6f). In contrast, no PTV RNA was detected in any tissues from the control group.

**Figure 7:**
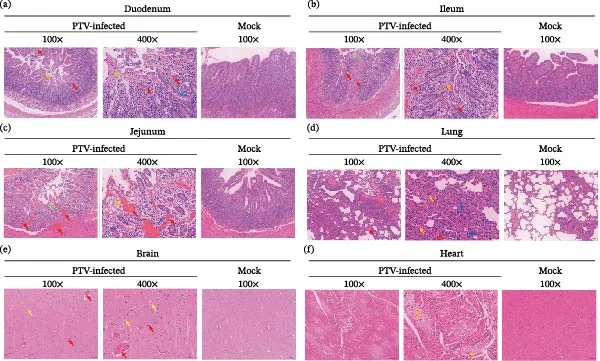
Histopathological analysis of major organs from Tibetan piglets infected with PTV‐SCgz‐01. Representative H&E‐stained sections of the duodenum (a), ileum (b), jejunum (c), lung (d), brain (e), and heart (f) are shown. For each organ, the left panel (100×) displays pathological lesions from an infected piglet, the middle panel (400×) provides a higher‐magnification detailed view, and the right panel (100×) shows the corresponding normal tissue from a mock‐infected control. The yellow arrows indicate necrotic cell debris in the small intestine, hyperplastic alveolar epithelial cells in the lung, neuronal degeneration in the brain, and myocardial fiber degeneration in the heart. The blue arrows indicate lymphocytes in the small intestine and fibroblasts in the lung. The red arrows indicate congested and dilated capillaries in the small intestine, lung, and brain. The green arrow indicates necrosis and lysis of the mucosal layer in the small intestine.

**Figure 8:**
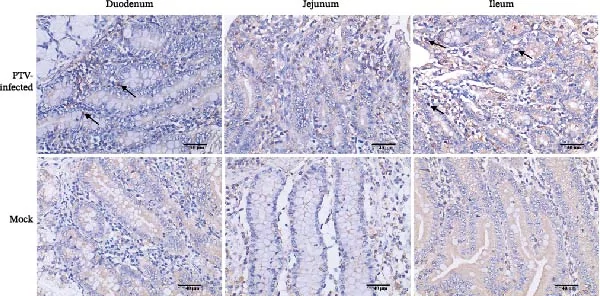
Segmental detection of PTV antigen in the small intestine by immunohistochemistry. Representative IHC images from PTV‐SCgz‐01‐infected piglets are shown. Viral antigens are readily detected in the duodenum and ileum, with less distribution in the jejunum. The black arrows indicate the PTV antigen. Scale bar = 40 μm.

Histopathological assessment of the challenged group revealed consistent and significant multiorgan pathology. The most severe pathological lesions were observed in the small intestine, which exhibited extensive villous necrosis and sloughing, a robust inflammatory cell infiltrate, and capillary dilation with congestion (Figure 7a–c). Pulmonary lesions were characterized by alveolar epithelial hyperplasia, interstitial fibrosis, and marked capillary congestion (Figure 7d). Cerebral sections demonstrated prominent neuronal degeneration accompanied by moderate capillary dilation and congestion (Figure 7e). Myocardial degeneration was evident in the heart (Figure 7f). The presence of PTV‐SCgz‐01 within these tissues was confirmed by IHC, which detected viral antigens mainly distributed in the duodenum and ileum, with less distribution in the jejunum (Figure 8). This specific intestinal tropism, coupled with the observed histopathology, demonstrates that PTV‐SCgz‐01 successfully infects and replicates within intestinal epithelial cells, causing extensive tissue damage in suckling piglets. All control tissues were histologically unremarkable.

## 4. Discussion

PTV was first identified in 1929 following a devastating outbreak of polioencephalomyelitis in the Czech Republic, with mortality exceeding 70% in affected herds. Although improved prevention measures have since led to the global predominance of low‐virulence, often subclinical strains [13, 14], PTV remains an economically important swine pathogen associated with enteric, respiratory, reproductive, and neurological diseases. In China, however, highly pathogenic variants have caused sporadic outbreaks in recent years [12, 21], posing persistent threats to swine health and production. Currently, epidemiological investigations on the PTV virus mainly focus on commercial domestic pigs, while data on PTV in Tibetan pigs remain limited. As a unique indigenous breed reared under high‐altitude free‐range conditions on the Tibetan plateau of southwest China, Tibetan pigs exhibit marked differences from domestic pigs in their genetic background and rearing system, representing a neglected host for PTV adaptation and evolution. Accordingly, this study provides a systematic investigation into the prevalence, genetic diversity, and pathogenicity of PTV in this distinct plateau pig population.

In China, epidemiological surveys showed that PTV circulates widely in swine herds, with the prevalence differing by region, breed, and management system. In low‐altitude domestic pigs, infection rates vary from 19.03% in Hunan Province [27] to 85.7% in Shanghai [13]. A serological study in northern China documented a PTV‐8 antibody positivity rate of 61.3% [19]. For indigenous two‐end‐black pigs in Jiangxi Province, the overall positivity rate of PTV reached 62.50%, with 90% of nursery pigs testing PTV‐positive [14]. In high‐altitude Tibetan pigs, PTV was detected in 141 out of 303 samples (positive rate: 46.53%), and the virus was detected on 19 out of 21 pig farms (positive farm rate: 90.5%). The positivity rate of PTV in this study is notably higher than the 20.8% previously reported in Tibetan pigs from Kangding [26], which may reflect the distinct sampling populations: all of our samples were collected from diarrheic Tibetan piglets, whereas 52% (50/96) of the samples in the previous report were derived from healthy fecal specimens. The high farm‐level prevalence (90.5%) suggests widespread distribution across the investigated farms and may be an important enteric pathogen in symptomatic Tibetan piglets. However, because all samples were obtained from piglets with diarrhea, the observed positivity rate likely reflects a symptomatic population and may overestimate the true prevalence in Tibetan pigs overall. Given that pigs infected with low‐virulence PTV strains exhibit only subclinical or mild clinical symptoms, further collection and testing of samples from Tibetan pigs without obvious clinical manifestations should be undertaken in this study to better reflect the true prevalence of PTV in this population.

Phylogenetic analyses based on the VP1 and P1 genes have been validated as reliable approaches for genotyping PTV [7, 9, 27]. Since the first PTV‐1 strain was isolated in Inner Mongolia in 2003 [17], multiple PTV genotypes have been identified in China. Early viral surveillance detected PTV‐8 in Jilin in 2009 [18], and subsequent studies identified PTV‐2, PTV‐4, PTV‐6, and PTV‐8 as the predominant genotypes circulating in Northeast China [19]. In 2015, Sun et al. [13] reported PTV‐4 as the most common genotype in Shanghai, followed by PTV‐8 and PTV‐10. A long‐term surveillance study conducted in Hunan between 2014 and 2017 revealed extensive viral diversity, with up to 20 PTV genotypes identified [20]. Similarly, based on VP1 sequencing, 11 distinct genotypes were characterized in Jiangxi [15]. In the present study, 22 PTV sequences obtained from Tibetan pigs were clustered into 12 known genotypes. Strikingly, five additional sequences formed three unclassified clusters, indicating high genetic diversity among PTV strains circulating in Tibetan pigs. To the best of our knowledge, this is the first report of *Teschovirus B* strains in Tibetan pigs.

Recombination is an important mechanism driving the evolution of picornaviruses [28, 29]. To date, however, documented recombination events among PTV strains remain limited, although recent studies have reported natural recombinants involving interserotypic and intragenotypic exchanges [4, 22, 23, 27, 30]. In this study, genomic analysis indicated that PTV‐SCgz‐01 is likely a natural recombinant derived from PTV‐4 and PTV‐6 subtypes, with parental strains originating from domestic pigs in Heilongjiang (HNMY) and Tibetan pigs in Sichuan (China/SWU‐ZG2/2018). Our findings suggest the possibility of cross‐regional viral transmission and genetic exchange between different swine hosts.

PTV infection affects pigs of all ages and is often subclinical, with young piglets being the most susceptible [31]. Most circulating PTV strains cause asymptomatic carriage or mild enteric signs; however, highly pathogenic variants have emerged sporadically across China in recent years. In 2016, a neurotropic PTV‐1 variant (CHN‐NP1‐2016) was identified in northeastern China and confirmed to induce nonsuppurative encephalitis and posterior paralysis in neonatal pigs [30]. In 2020, the PTV‐2 strain HeNZ1 caused a severe outbreak on a large‐scale farm in Henan Province, leading to typical neurological manifestations and 38% mortality in suckling piglets [21]. A recent study reported a severe PTV outbreak in Gansu Province characterized by severe diarrhea, with mortality exceeding 60% in suckling pigs and ~20% in fattening pigs [12]. In the present study, the newly identified isolate PTV‐SCgz‐01 caused severe watery diarrhea, pronounced neurological signs, and 40% mortality (2/5) in 17‐day‐old Tibetan piglets. Of note, histopathological examination revealed widespread multiorgan lesions and broad tissue tropism. A limitation of the animal study is that potential bacterial enteric pathogens were not screened in the experimental piglets before the PTV challenge. Although the animals were obtained from a diarrhea‐free farm and were negative for PTV and several viral enteric pathogens, the contribution of occult bacterial infection cannot be completely excluded. Future challenge studies should include common bacterial screening (e.g., *Escherichia coli*, *Salmonella*, and *Clostridium perfringens*) to further strengthen the pathogenicity assessment. Our findings indicate that the newly emerged PTV‐SCgz‐01 strain showed strong pathogenic potential in experimentally infected 17‐day‐old Tibetan piglets. However, the pathogenicity evaluation of the PTV isolate was based on a small number of piglets, a single challenge dose, and a single age group. Therefore, further studies are required to fully characterize the virulence of PTV‐SCgz‐01 under natural infection conditions.

## 5. Conclusions

In summary, our study revealed the high prevalence and abundant genetic diversity of PTVs in Tibetan pigs, extending previous findings in this high‐altitude pig population. Furthermore, a natural recombinant (PTV‐SCgz‐01) between PTV‐4 and PTV‐6 was isolated and characterized. Animal experiments demonstrated that the newly emerged isolate has strong pathogenic potential to 17‐day‐old Tibetan piglets, causing severe watery diarrhea, with a high mortality rate of 40%. Our findings provide systematic molecular epidemiological data on PTVs in Tibetan pigs and underscore the urgent need for viral surveillance and control measures within this unique pig population.

## Author Contributions

**Haohao Lu**: data curation, formal analysis, visualization, writing – original draft. **Min Zhang**: resources, supervision, writing – review and editing. **Danjiao Yang**: investigation, resources. **Xue Gao**: data curation. **Shuo Feng**: investigation. **Yiwen Pei**: software, validation. **Zhidong Zhang**: methodology. **Long Zhou**: conceptualization, funding acquisition, project administration, resources, writing – review and editing.

## Funding

This work was supported by the Fundamental Research Funds for the Central Universities, the Southwest Minzu University (Grant ZYN2026036), the Sichuan Science and Technology Program (Grant 2025ZNSFSC0192), and the Ganzi Prefecture Science and Technology Plan Project (Grant 25kjjh0013).

## Ethics Statement

The animal study was approved by the Animal Welfare and Ethic Committee of the Institute of Animal Science of Ganzi Tibetan Autonomous Prefecture with the Reference Number IASGTAP2025‐021.

## Conflicts of Interest

The authors declare no conflicts of interest.

## Supporting Information

Additional supporting information can be found online in the Supporting Information section.

## Supporting information


**Supporting Information** Figure S1: Comparative VP1 sequence analysis of five unclassified PTV strains against 22 reference PTV genotypes. (a–c) Multiple amino‐acid sequence alignments of the VP1 protein. (d) Nucleotides and amino acids homologies between the five undefined genotypes and other recognized PTV strains. (e) Detailed information of the 22 PTV reference strains used in this study.

## Data Availability

The data used to support the findings of this study are included within the article. The PTV nucleotide sequence data of this study were submitted to the GenBank database (http://www.ncbi.nlm.nih.gov/genbank/) under Accession Numbers PX625739 and PX625740–PX625766. The data that support the findings of this study are available in the Supporting Information of this article.
