# Supplementary material for: Molecular Epidemiology and Pathogenicity Evaluation of Porcine Teschovirus in Tibetan Pigs on the Qinghai–Tibet Plateau of China
Source: Transbound Emerg Dis. 2026 Sep 7;2026:8803438. doi: 10.1155/tbed/8803438 (PMC13549367; doi:10.1155/tbed/8803438)
Supplement: Supplementary file 1 [file TBED-2026-8803438-s001.docx]

**Figure S1.** Comparative VP1 sequence analysis of five unclassified PTV strains against 22 reference PTV genotypes. (a‑c) Multiple amino‑acid sequence alignments of the VP1 protein. (a) Alignment of SCgz03 versus reference strains; (b) Alignment of SCgz25 versus reference strains; (c) Alignment of SCgz18, SCgz22 and SCgz26 versus reference strains. (d) Nucleotides and amino acids homologies between the five undefined genotypes and other recognized PTV strains. (e) Detailed information of the 22 PTV reference strains used in this study.
